# Oscillatory Dynamics of Cell Cycle Proteins in Single Yeast Cells Analyzed by Imaging Cytometry

**DOI:** 10.1371/journal.pone.0026272

**Published:** 2011-10-26

**Authors:** David A. Ball, Julie Marchand, Magaly Poulet, William T. Baumann, Katherine C. Chen, John J. Tyson, Jean Peccoud

**Affiliations:** 1 Virginia Bioinformatics Institute, Virginia Tech, Blacksburg, Virginia, United States of America; 2 Department of Electrical and Computer Engineering, Virginia Tech, Blacksburg, Virginia, United States of America; 3 Department of Biological Sciences, Virginia Tech, Blacksburg, Virginia, United States of America; 4 Institute for Critical Technology and Applied Science Center for Systems Biology of Engineered Tissues, Virginia Tech, Blacksburg, Virginia, United States of America; Duke University Medical Center, United States of America

## Abstract

Progression through the cell division cycle is orchestrated by a complex network of interacting genes and proteins. Some of these proteins are known to fluctuate periodically during the cell cycle, but a systematic study of the fluctuations of a broad sample of cell-cycle proteins has not been made until now. Using time-lapse fluorescence microscopy, we profiled 16 strains of budding yeast, each containing GFP fused to a single gene involved in cell cycle regulation. The dynamics of protein abundance and localization were characterized by extracting the amplitude, period, and other indicators from a series of images. Oscillations of protein abundance could clearly be identified for Cdc15, Clb2, Cln1, Cln2, Mcm1, Net1, Sic1, and Whi5. The period of oscillation of the fluorescently tagged proteins is generally in good agreement with the inter-bud time. The very strong oscillations of Net1 and Mcm1 expression are remarkable since little is known about the temporal expression of these genes. By collecting data from large samples of single cells, we quantified some aspects of cell-to-cell variability due presumably to intrinsic and extrinsic noise affecting the cell cycle.

## Introduction

The cell division cycle is the sequence of events whereby a living cell replicates its components and divides them between two daughter cells, so that each daughter receives the information and machinery necessary to repeat the process. Progression through the cell cycle is governed by a complex but precise molecular mechanism relying on checkpoints to ensure that every newborn cell receives one complete set of chromosomes [1]. Although the sequence of events is very tightly controlled, the time taken to progress through each stage of the cell cycle may vary considerably from cell to cell. Modelers have recognized the need to incorporate this cell-to-cell variability into their models, and have started to transform their deterministic models into stochastic versions [2], [3]. In a recent paper, we used stochastic modeling and single-cell microscopy to characterize a budding yeast mutant that exhibits stochastic fluctuations between cell division and cell cycle arrest when grown on alternative carbon sources (e.g., raffinose) that support slower growth rates than glucose [4].

Previous research into the expression of genes controlling progression through the eukaryotic cell cycle has heavily relied on bulk measurements, such as western (and northern) blots and micro-arrays, on populations of cells that have been synchronized by some strong perturbation, for examples see the experimental data used in the development of the model of Chen et al [5].

It has been argued that batch-culture synchronization methods are incapable of creating reliably synchronous populations of cells [6], [7]. Proponents of these methods point to the vast amounts of microarray data that have been collected to show that, although not perfect, synchronization has revealed many molecular features of the cell cycle that were previously unknown [8], [9]. In any case, one thing that Cooper and Spellman do agree on is that synchronization introduces artifacts that can be difficult to judge. In addition, bulk measurements largely ignore subtle differences between individual cells that arise due to molecular noise [10], [11]. However, recent advances, such as the introduction of fluorescent proteins optimized for various organisms [12] and the development of automated microscopy, have allowed the community to begin to re-examine this complex gene network at the single-cell level [13]–[25].

**Figure 1:**
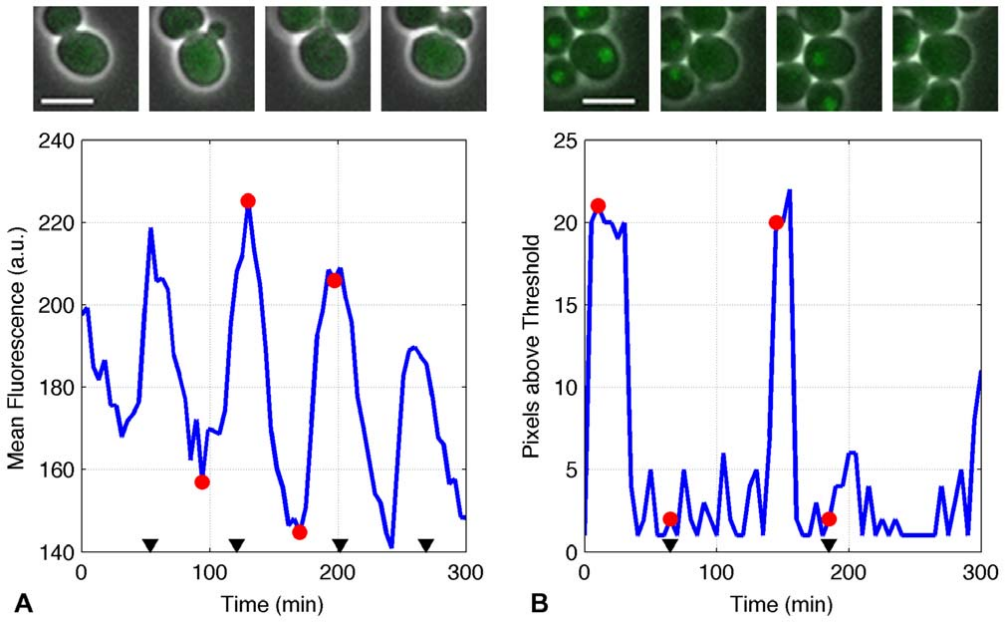
Indicators of protein abundance and protein localization. (A) Comparison between Cln2-GFP images and indication of protein abundance. Here the cell in the center of the image exhibits a noticeable difference in cell fluorescence between images 1 and 3 (low) and images 2 and 4 (high). (B) Comparison between Whi5-GFP images and the indicator of protein localization. On images 1 and 3, when looking at the cell in the center of the images, it is possible to recognize a subcellular structure where more fluorescence is concentrated than in the other regions of the cells. In both parts, the top of the figure shows four images taken over two cell cycles, while the bottom of the figure shows the indicator of protein abundance (A) or fluorescence localization (number of pixels above the threshold) (B). The red circles correspond to the time-points of the four images. The black triangles indicate budding events. The scale bars in the images indicate 5 µm.

Different groups have used these tools to explore various aspects of the cell cycle in individual yeast cells. For example, Tully et al. used live-cell imaging to examine the role of the anaphase-promoting complex (APC) in cytokinesis by use of GFP fusions of the actomyosin ring component Iqg1 [23]. Fred Cross's group has used live-cell imaging of fluorescently tagged genes to investigate protein dynamics at the G1-S transition [14] and at mitotic exit [22], [25]. More commonly, though, fluorescently labeled proteins are used as staging markers indicative of specific events in the cell cycle. Tagging Myo1 for instance facilitates the detection of bud emergence as this protein concentrates in the bud-neck at this particular stage [16]. Such methods have been extremely useful in determining the roles that noise plays in cell cycle progression [16], and in analyzing how the cell cycle is perturbed in various mutant strains of budding yeast [15], [18], [20], [21], [24]. Rather than using GFP-tagged proteins as timers of cell cycle events in wild-type and mutant cells, we are more interested in their use as reporters of gene expression levels. In this paper, using a representative selection of 16 GFP-tagged cell cycle genes in budding yeast, we provide a broad assessment of the temporal patterns of protein abundance and localization during the cell cycle and of the magnitude of noise affecting these proteins. Using time-lapse microscopy we measured the fluorescence signals of individual cells through 4,835 cell cycles. We developed custom signal processing, data aggregation, and statistical analysis methods to estimate the period, amplitude, and phase of oscillation in the abundance profiles of these 16 cell cycle regulatory proteins. We also monitored the localization of these proteins throughout the cell cycle. Our analysis shows that there are noticeable differences in the noise affecting different proteins. Some appear to be more tightly controlled than others, and all are more noisy than the cell division process itself, suggesting that the cell cycle is somewhat shielded from the fluctuations affecting individual proteins. Examples of both protein abundance and protein localization analyses are shown in Figure 1. We compare our single-cell, single-protein observations with protein dynamics reported in the experimental literature, which are summarized in Table 1. Statistically significant oscillations were observed in the abundance of Net1 and Mcm1, an observation that has not been previously reported in the literature.

**Table 1:** Known features of proteins involved in the *S. cerevisiae* cell division cycle.

Gene	Protein Role[45]	Protein Localization[43]	Oscillation of mRNA[27]	Mean Number[46]
*BCK2*	Bck2 is a Cln-independent activator of *CLN1,2* expression [24].	no	no	Not visualized
*BUB2*	Mitotic exit network regulator blocks cell cycle progression before anaphase in response to spindle misalignment and kinetochore damage.	bud (Anaphase), otherwise spindle-poles [47]	no	Not visualized
*CDC6*	Component of the pre-replicative complex and a stoichiometric inhibitor of Cdc28-Clb kinase.	cytoplasm (S-phase to end of anaphase), otherwise nucleus [48]	high: late M-phase	Not visualized
*CDC15*	Protein kinase necessary for mitotic exit.	spindle-poles	no	238
*CDC20*	Auxiliary component of the anaphase-promoting complex; promotes degradation of Clb's and Pds1.	no	high: M-phase	Not visualized
*CLB2*	B-type cyclin involved in progression through M phase; accumulates during G2 and M, and is degraded during anaphase and telophase by ubiquitin-mediated proteolysis	nucleus	high: G2	339
*CLN1*	G1 cyclin involved in the Start transition (late G1) and bud emergence.	no	high: late G1	319
*CLN2*	G1 cyclin involved in the Start transition (late G1) and bud emergence.	no	high: late G1	1270
*ESP1*	Separase, a caspase-like cysteine protease that promotes sister chromatid separation by cleaving the cohesin rings that bind sister chromatids together at centromeres	nucleus (metaphase-anaphase transition) [49]	high: S-phase	Low signal
*LTE1*	LTE1 is essential for termination of M phase at low temperatures, along with TEM1, and CDC15 (5), all part of the mitotic exit network	cytoplasm	no	304
*MAD2*	Spindle assembly checkpoint protein; sequesters and prevents Cdc20 from activating APC	no	no	1110
*MCM1*	Transcription factor for Clb2 and Cdc20	nucleus	no	8970
*NET1*	Core subunit of the RENT complex, which is involved in nucleolar silencing and telophase exit	nucleolus	no	1590
*SIC1*	Inhibitor of Cdc28-Clb kinase complexes, active in G1 phase	no	high: end of mitosis & G1	768
*TEM1*	GTPase component of the mitotic exit network [1]	spindle pole	high: G2	573
*WHI5*	Repressor of G1 transcription, binds to SCB binding factor (SBF) in early G1	nucleus (late mitosis) leaves during G1 [19]	high: S-phase	1440

## Results

### Single cell analysis

#### Protein abundance

**Figure 2:**
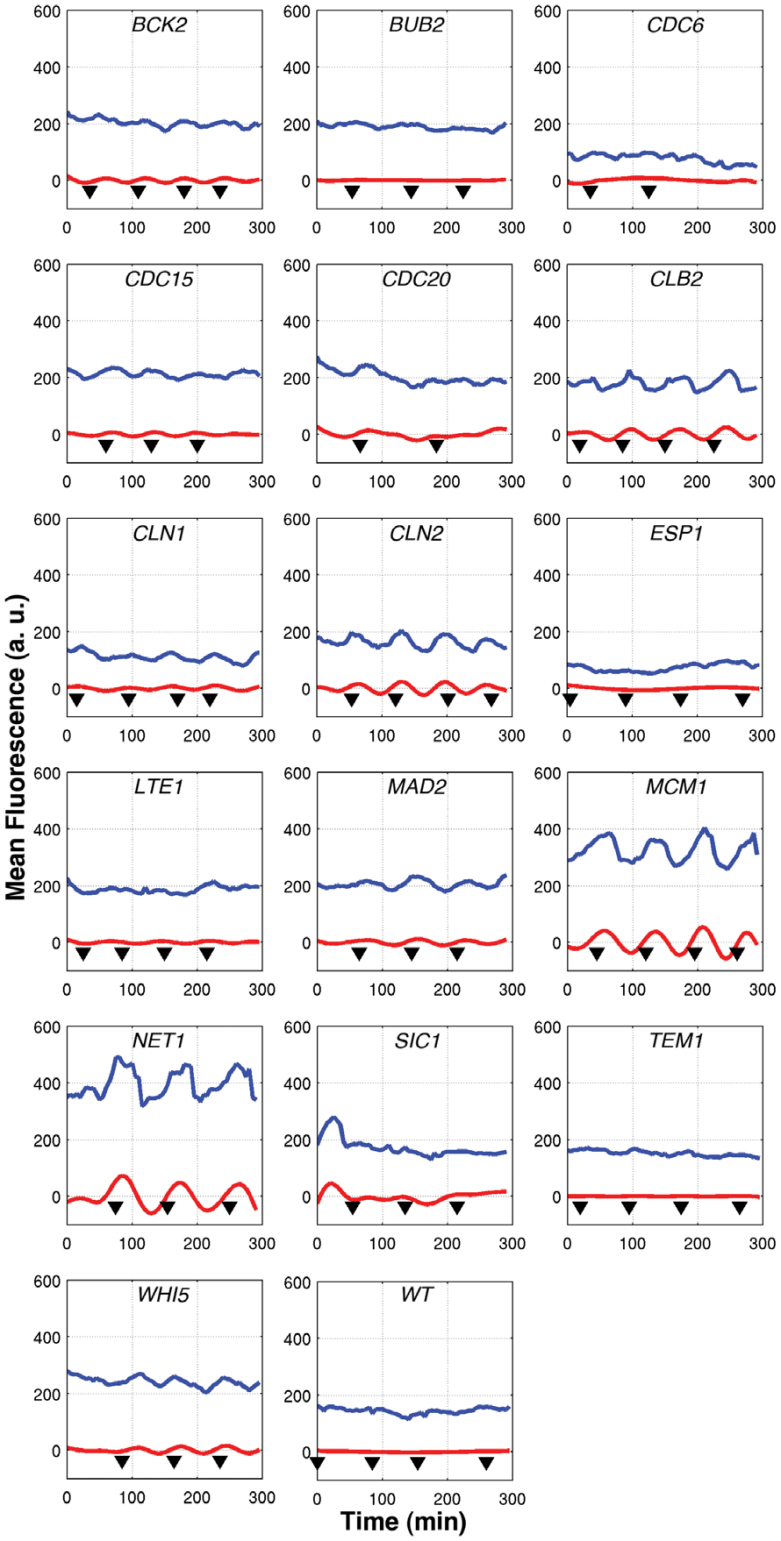
Mean cell fluorescence for individual cells. The measured mean fluorescence is plotted (blue line) along with the filtered signal (red line) for a single cell expressing GFP as a fusion with the indicated cell-cycle regulator. Filtering removes all high-frequency noise, as well as the DC offset, leaving signals that have a zero mean. Budding events are indicated by black triangles.

First, we tracked the time evolution of cell fluorescence over several cell cycles (Figure 1A). The mean fluorescence of pixels within a cell in a specific frame is used as an indicator of the tagged protein concentration at a particular instant of time. By tracking cells over several frames, it is possible to reconstruct the time evolution of the fluorescence over several cell division cycles spanning several hours. In order to smooth the time-courses of individual cells, non-physiological oscillation frequencies were filtered out of the trajectories. High-frequency noise components with periods of less than 50 minutes, which could originate from sources such as the camera read noise, or segmentation errors, were removed. The unfiltered and filtered time-course of a typical cell for each of the 16 yeast strains are shown in Figure 2, along with a wild-type (WT) control cell not labeled with GFP. The timing of the budding event was manually annotated for each cell and is reported as a black triangle on the x-axis of fluorescence profiles in Figure 2. This series of data extraction and signal processing steps generates a clean signal consistent with some known aspects of cell cycle dynamics. For instance the fluorescence signals collected on the Cln2 and Clb2 tagged strains are well correlated with the cell budding event.

#### Protein localization

**Figure 3:**
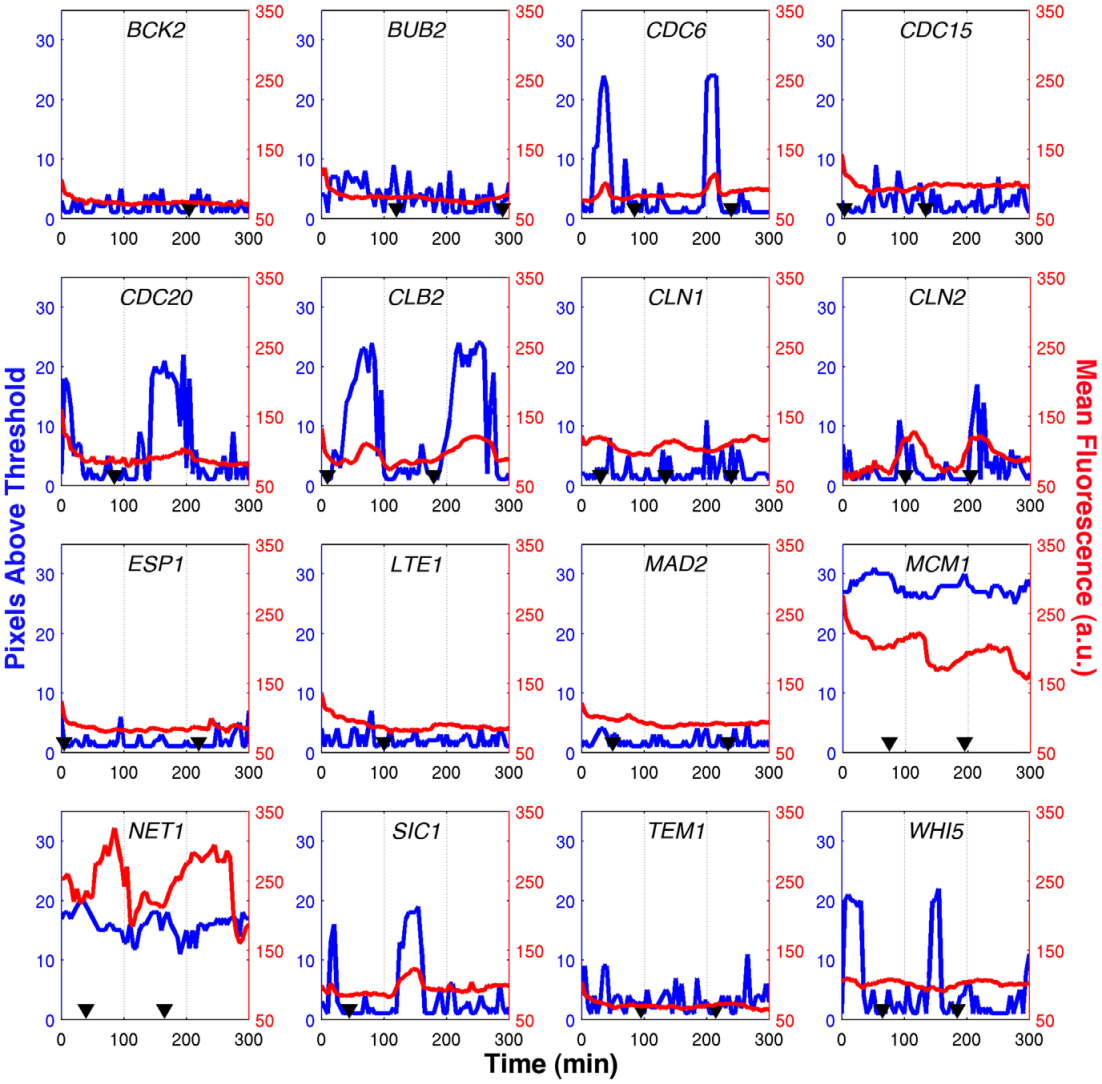
Localization of GFP-tagged proteins. Z-stacks of cells expressing a GFP fusion of the indicated protein were projected to a single image and analyzed to determine changes in localization through the cell cycle. The number of connected pixels within the 95^th^ percentile (blue curve) was used as a metric to calculate the degree of localization, with a higher number indicating a larger area of bright pixels. A typical cell from each strain is shown, along with that cell's mean fluorescence over the entire cell (red curve) and the manually annotated budding events (black triangles). Note that the increased light exposure (∼20×) resulted in much slower cell-division compared to the single-plane experiments described.

Although a single fluorescently tagged protein cannot be used to unambiguously determine the organelle in which the protein is located, it is possible to determine the area (number of pixels) to which a protein is confined, and how this area changes over time (for example, see Figure 1B). To reduce the effects of organelle motion in and out of focus, these measurements were conducted by collecting images at multiple focal planes for each sample. These multiple focal planes were then combined using a maximum z-projection (see Materials and Methods). To quantify protein localization, we propose an estimator (‘pixels above threshold’, see Materials and Methods) and plot this estimator as the blue line in Figure 3. The small numbers of pixels above threshold observed for Bck2, Bub2, Cdc15, Esp1, Lte1, Mad2 and Tem1 indicate that these proteins are uniformly distributed throughout the cell, with only a few bright pixels located next to each other by chance. The patterns observed for Mcm1 and Net1 indicate that these proteins remain extremely localized throughout the cell cycle. Cdc6, Cdc20, Clb2, Sic1, and Whi5 exhibit a couple of sharp bursts over a period of 300 min. In other words, these proteins are distributed evenly throughout the cell volume during most of the cell cycle except during a short period of time (once per cycle), when they concentrate in one location in the cell.

The red plots on Figure 3 report the mean cell fluorescence as described above except that it is computed over 20 focal planes. For some proteins (Cdc6, Clb2, Cln2, Sic1), subtle correlations between changes of protein abundance and protein localization can be observed while the changes of localization observed for Cdc20 and Whi5 occur without any observable changes of protein abundance. Net1 and Mcm1 exhibit complex asymmetric patterns indicating a slow accumulation of protein followed by very rapid decrease in protein abundance.

Unfortunately, the increased light exposure resulting from the observation of multiple focal planes negatively impacted cell growth. As a result only a few cells for each cell line could be observed. It has not been possible to properly assess the fluctuation of protein localization statistics from these small samples.

### Population-level statistics

In order to characterize the stochastic dynamics of the cell cycle, we observed 4,835 individual cell cycles and reduce this large data set by extracting the amplitude and period of the fluorescence signals and its relation to budding times. The statistical distribution of these random variables is likely to provide an indication of the global structure of the noise affecting the cell cycle.

#### Budding events

To determine if the fusion of GFP to any of the investigated proteins was detrimental to the function of that protein, we characterized the division times of each cell by calculating the time between budding events for mother cells (inter-bud time), and the time from a daughter's emergence as a bud to its first bud (birth-to-bud time) for each of the strains (Table 2). For most of the strains in this study, the observed cell cycle distributions agree well with each other as well as with the distributions of wild-type cells reported in the literature for both mothers and daughters [5]. With an inter-bud time of 132.2 min compared to 90.0 min for the WT, the *CDC20-GFP* strain is the only one that exhibits a noticeable phenotypic effect that may result from an alteration of the function of the Cdc20 protein by GFP tagging. Hence, we do not include Cdc20 in our further analysis of protein fluctuations.

**Table 2:** Extracted oscillation parameters and statistics of inter-bud times (mothers), and birth-to-bud times (daughters) for GFP fusions of the indicated proteins.

		Amplitude		Protein vs. WT	Period (min)		Mother inter-bud times			Daughters birth-to-bud times		
tagged gene	# cycles	Median	CV	p-value (t-test)	Median	CV	# cycles	Median	CV	# cycles	Median	CV
***BCK2***	211	13.48	0.83	0.046	64.99	0.50	139	74.99	0.26	109	109.99	0.25
***BUB2***	242	12.59	0.75	0.133	75.00	0.42	125	80.00	0.28	105	110.01	0.24
***CDC6***	93	11.47	0.94	0.434	85.01	0.52	83	80.01	0.30	65	105.01	0.29
***CDC15*** *	125	17.77	0.73	<0.0001	75.00	0.48	129	75.00	0.19	85	100.00	0.27
***CDC20*** *	26	20.07	0.66	<0.0001	85.16	0.42	20	132.23	0.22	21	129.98	0.20
***CLB2*** *	254	29.78	0.47	<0.0001	80.29	0.32	111	80.29	0.32	101	100.36	0.22
***CLN1*** *	185	19.33	0.60	<0.0001	79.99	0.29	96	84.99	0.26	80	109.99	0.22
***CLN2*** *	63	39.99	0.46	<0.0001	80.68	0.46	64	80.68	0.28	56	118.78	0.20
***ESP1***	95	13.22	0.96	0.071	90.00	0.41	100	85.00	0.24	70	120.00	0.26
***LTE1***	69	13.42	0.83	0.049	90.00	0.51	103	80.00	0.25	67	105.00	0.26
***MAD2***	81	11.17	0.80	0.954	75.00	0.41	56	85.00	0.39	43	115.01	0.27
***MCM1*** *	467	65.33	0.41	<0.0001	75.27	0.34	181	75.27	0.27	151	105.38	0.24
***NET1*** *	114	74.87	0.93	<0.0001	80.00	0.32	53	80.00	0.24	43	115.01	0.18
***SIC1*** *	58	34.08	0.90	<0.0001	105.01	0.48	60	80.00	0.37	50	112.51	0.27
***TEM1***	53	14.38	0.83	0.687	74.98	0.52	31	79.98	0.29	24	117.47	0.37
***WHI5*** *	129	29.30	0.56	<0.0001	80.00	0.48	64	75.00	0.22	67	105.00	0.26
***WT***	107	11.83	0.97	1	84.98	0.41	138	89.98	0.31	116	99.97	0.26

* Genes with fluorescence oscillations statistically different from the WT.

#### Protein abundance in absolute time

**Figure 4:**
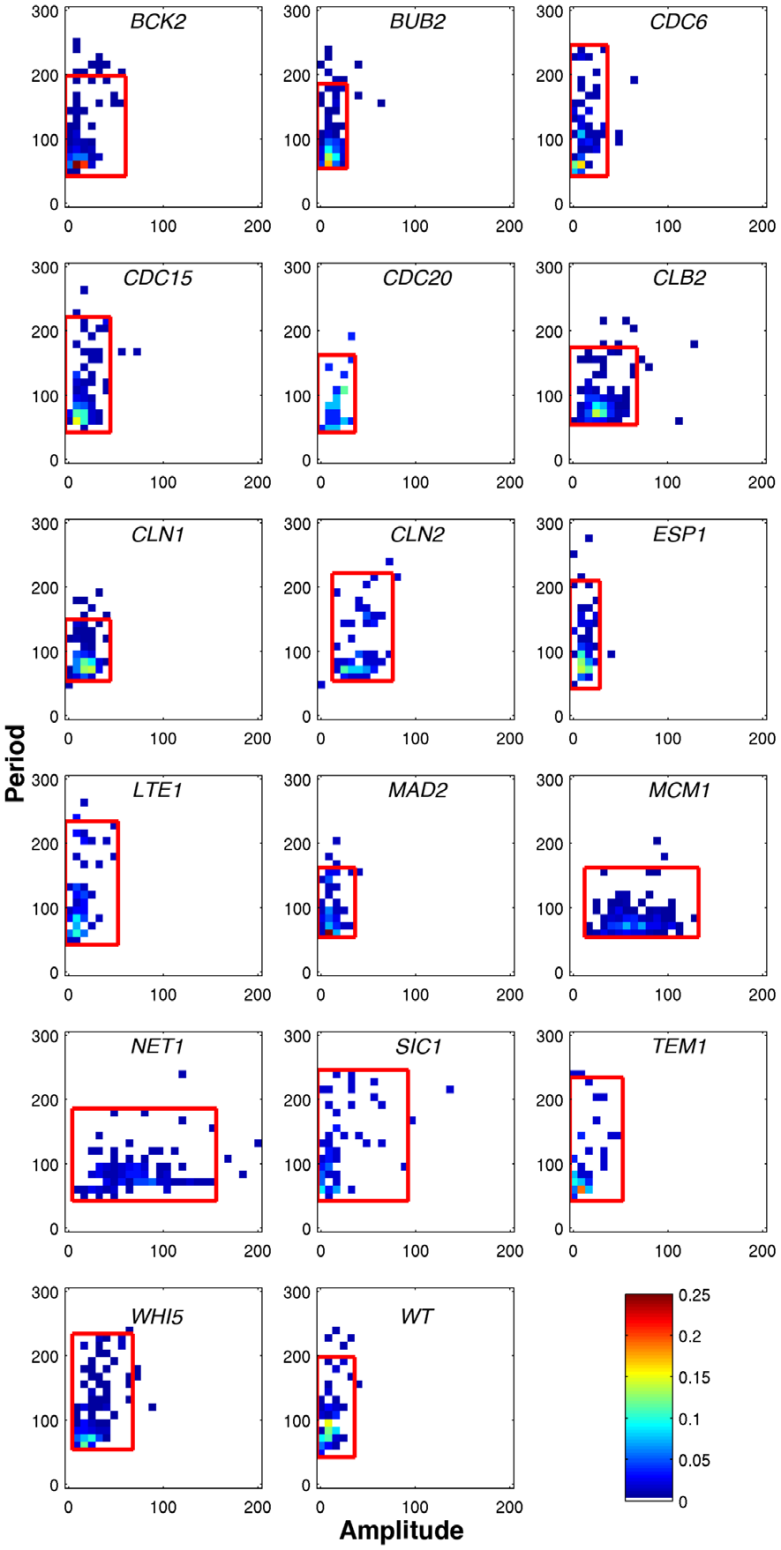
Distributions of amplitude and period estimates for gene expression for 16 GFP-tagged strains and WT. Red boxes enclose 95% of all cells. Low-pass Butterworth filtering removes all oscillations with non-physiological periods, which leaves only periods greater than 50 min. Using a t-test, we compared the amplitude distribution (x-axis) for each strain with that of wild-type (wt) cells to determine if the protein level exhibits noticeable oscillations. The color of each bin represents the fraction of cell cycles that fall in that bin, indicated by the colorbar in the lower right.

The dynamics of the mean fluorescence observed on individual cells was characterized by using the peak-to-peak times of the filtered fluorescence signal to measure the period of fluorescence fluctuations, and the difference between the maximum and minimum fluorescence values to determine the amplitude of fluorescence fluctuations. The period and amplitude of fluorescence oscillations were aggregated over multiple cell cycles collected over several individual cells, and are shown in Figure 4. The medians and coefficients of variation of the mean fluorescence period and amplitude for all the 17 strains are reported in Table 2.

The joint distributions of amplitudes and periods can be represented by heat maps that give a visual indication of the variability of protein abundance over hundreds of cell cycles (Figure 4). This figure highlights the differences in the noise affecting the dynamics of these 16 proteins. For Bub2, Clb2, Cln1, and Mad2, the fluctuations of the period and amplitude are narrowly controlled. In other cases, one parameter is better controlled than the other. Mcm1 and Net1 have narrow period distributions but much wider amplitude distributions whereas Bck2, Cdc6, Cdc15, Cln2, Esp1, Lte1, Tem1, and Whi5 have a narrow amplitude distribution but a wider period distribution. Finally, Sic1 exhibits broad fluctuations of both period and amplitude of fluorescence oscillations. Since these plots correspond to experiments that were conducted on different days with different cell cultures, it is possible that the differences observed reflect some uncontrolled experimental parameters. In order to rule out this possibility, all strains were profiled twice several months apart, and the results were qualitatively similar even though quantitative differences were observed.

To determine whether a GFP-tagged protein is exhibiting statistically significant oscillations in abundance during the cell cycle, we use an unequal variance t-test to compare the tagged protein's amplitude distribution against the background fluorescence fluctuations observed in the untagged WT strain from which the tagged strains were derived. At the 1% significance level (p-value<0.01 in Table 2), we identify Cdc15, Clb2, Cln1, Cln2, Mcm1, Net1, Sic1, and Whi5 as oscillatory proteins (they are marked with an * in Table 2).

For most of the strains exhibiting oscillatory fluorescence, the period of fluorescence is in good agreement with the mother inter-bud time, which is expected, as the software follows the mother cell. Sic1 is a marginal case. The tagged strain proliferates normally (median inter-bud time of mother cells = 80 min), but the median period of fluorescence oscillations is significantly longer (105 min). We choose to retain Sic1 in our further considerations of oscillatory proteins.

#### Protein abundance in normalized time

**Figure 5:**
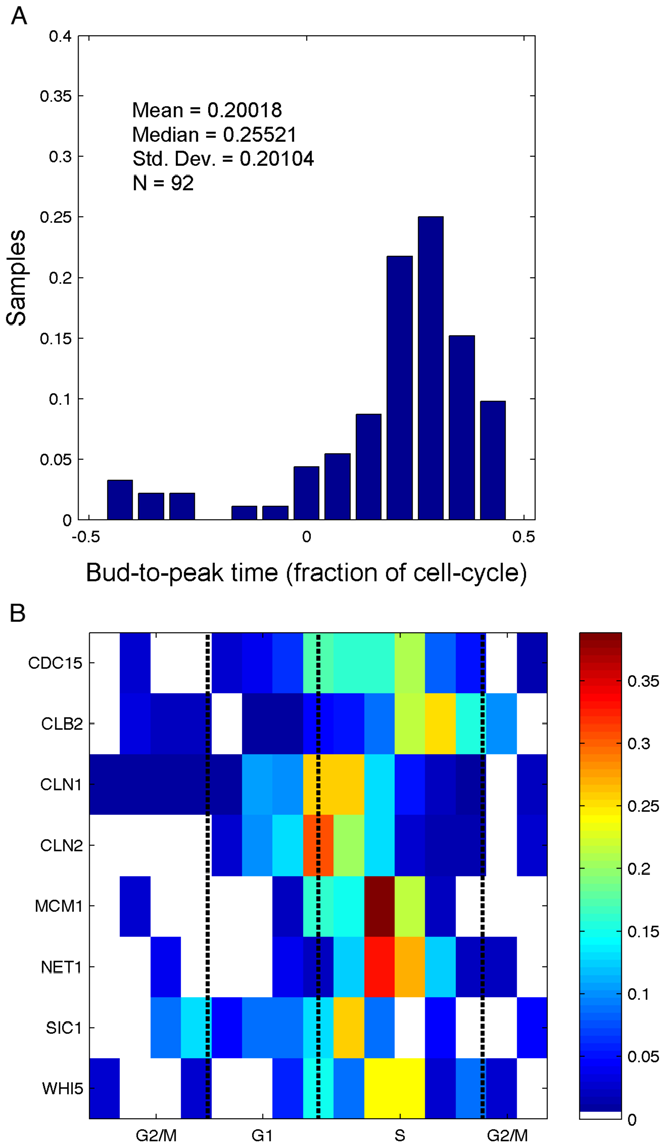
Phase of peak expression in cell-cycle normalized time. (A) Distribution of the time between bud emergence and peak fluorescence for the CLB2-GFP strain. (B) Distributions of all GFP-tagged strains, with color representing fraction of cells comparable to the height of each bin displayed in (A). Cell-cycle stages are based on the relative length of each stage from [26], and assuming that bud emergence occurs at the G1-S transition. The boundaries between the different stages are represented by the dashed vertical lines. Because of the difficulty in identifying all budding events, the bud-to-peak time value is found by locating the closest peak in the fluorescence time-course to each identified budding event.

In order to determine the point in the cell cycle that peak expression occurs for each protein, we related fluorescence data to manually annotated budding events used for estimating the inter-bud and birth-to-bud times as described above. However, due to cells growing out of the plane of focus, it is not possible to identify all budding events. The phase of the protein expression was taken as the time between a bud emerging and the closest fluorescence peak. For instance, the distribution of bud-to-peak fluorescence times observed on the CLB2-GFP cell line shows that Clb2 abundance generally peaks roughly one quarter of a cell-cycle after bud emergence (Figure 5A). A similar analysis was performed for eight oscillating genes identified above (Cdc15, Clb2, Cln1, Cln2, Mcm1, Net1, Sic1, and Whi5). Results are reported in Figure 5B as heat maps indicating the distribution of the peak of fluorescence. In this figure, the stages of the cell cycle were determined using the information in [26], with the budding event assumed to occur at the G1-S transition. The data analysis procedure was verified to not introduce any unwanted artifacts by its use on simulated cells with fluorescence trajectories composed of purely white noise (Appendix S1).

It is interesting to compare (Table 3) the distribution of peak fluorescence times with the oscillatory dynamics of mRNA [27]–[29]. We compare our times of peak protein abundance to the peaks in mRNA levels reported by Gauthier et al [27], as their analyses includes the experiments of Pramila et al [28] as well as Spellman et al [29], and their results are presented in a searchable web database (www.cyclebase.org). Most of the genes we identified as oscillatory at the protein level also exhibit transcriptional oscillations (*CLB2*, *CLN1*, *CLN2*, *SIC1*, and *WHI5*). The peak in protein fluorescence coincides with the reported peak in mRNA level for *CLN1*, *CLN2*, and *WHI5*, but not for *CLB2* and *SIC1*. However, three of the genes oscillating at the protein level (*CDC15*, *MCM1*, and *NET1*) are not oscillating at the transcriptional level.

**Table 3:** Phase of fluorescence signal with respect to bud emergence, and comparison with mRNA peak.

Protein	Mean Protein Peak (fraction of cell cycle)	mRNA Peak [27] (fraction of cell cycle)	Difference
Cdc15	0.098	N/A	N/A
Clb2	0.200	0.410	−0.210
Cln1	0.025	0.000	0.025
Cln2	0.031	−0.030	0.061
Mcm1	0.103	N/A	N/A
Net1	0.151	N/A	N/A
Sic1	−0.032	−0.210	0.178
Whi5	0.119	0.090	0.029

For many of these proteins, time-courses of protein expression have been previously characterized by Western blots of synchronized cultures. Bck2 fluctuations during the yeast cell cycle have not been much studied; our results indicate that it is not a periodic protein. Similar to the single cell data presented here, Fraschini et al found the abundance of Bub2 to be constant during the 150 minutes following release from α-factor block in G1 [30]. Cdc6 was shown to be high in late M and early G1, and low otherwise [31]. Although we did not observe this in single plane fluorescence measurements, a similar pattern was observed in the z-stack reported in Figure 3. Jaspersen et al demonstrated that Cdc15 abundance is constant through the cell cycle after α-factor synchronization [32]. In contrast, we found the fluctuations in Cdc15-GFP fluorescence to be significantly different from that of untagged cells. Clb2 is known to peak in abundance at mitosis [33] but in our experiments, we find the peak fluorescence of Clb2-GFP to occur in late S phase. Tyers et al showed that Cln1 and Cln2 reach their peak abundances just after START [34], which is consistent with the observations presented here. Esp1 is another protein whose abundance is known to oscillate, with a distinct trough in G1 phase [35]. However, any fluorescence fluctuations in the ESP1-GFP strain were insignificant when compared to untagged cells. Our observations of GFP-tagged strains are consistent with literature reports that protein abundance is constant for Lte1 [36] and for Mad2 [37]. Mcm1 is reported to be non-oscillatory at the transcriptional level [29] but no reference reporting the evolution of protein abundance could be found. Our data shows that Mcm1 is always highly localized in the nucleus, and we see clear evidence of a sharp drop in mean fluorescence around the time of cell division The abundance of Net1 was measured to be constant throughout the cell cycle [38]. However, we see clear oscillations that peak, on average, in S phase. Sic1 abundance peaks in G1 and decreases at START [39]. We observe clear oscillations in Sic1-GFP fluorescence but at a later stage, shortly after START. The abundance of Tem1 is low in G1 and early S phases, begins to accumulate in M phase, and peaks during telophase [36] but our observations of fluctuations in Tem1-GFP fluorescence are indistinguishable from untagged cells. The visual inspection of images of GFP-tagged Whi5 did not reveal any obvious oscillation of protein abundance [40]. Our data set is consistent with these results as oscillations of GFP-tagged Whi5 are mild and barely detectable by visual inspection (Figure 2). However, the statistical analysis of the dataset uncovered evidence of oscillations peaking in S phase (Figure 5B). In agreement with the results of [40], we see that Whi5 is strongly localized in the nucleus in G1 phase (Figure 3). The relative expression of Sic1 and Whi5 (Figure 5B) also appears consistent with the results reported by Constanzo [40].

## Discussion

It is natural to compare the results presented in this report with current models describing the function, localization, and expression dynamics of the proteins studied in these experiments.

The set of genes analyzed here were chosen according to several criteria. In order to make a broad assessment of the effects of noise on cell cycle progression, we wanted to include in the experimental design as many cell cycle genes as practical. To ensure consistency and reproducibility, we chose to use GFP-tagged strains from an existing library. Some genes important for cell cycle regulation are not available in the library (e.g., *CLN3*, *CLB5*, *SWE1*). The genotype of the *CDC14-GFP* and *SWI5-GFP* strains indicate that the GFP tag is probably inserted in a different genomic location. We have therefore excluded these two important strains from this report.

It is important to remember that the criterion used to detect oscillations of protein abundance is based on a statistical comparison with the observed fluctuations of cell autofluorescence throughout the cell division cycle. As a result, for those proteins that show no evidence of periodicity by this criterion (namely, Bck2, Bub2, Cdc6, Esp1, Lte1, Mad2, and Tem1) it is not possible to decide whether they are present at constant levels throughout the cell cycle or oscillate with amplitudes smaller than the cell autofluorescence. The low levels of fluorescence that we observe for these GFP-tagged cell cycle proteins are actually puzzling. The weak signals may reflect the low abundances of these proteins. It is also possible that in some of the strains, the fluorescent domain is degraded rapidly compared to its maturation time because of the rapid turnover of the protein to which it is attached.

Our fluorescence measurements might also be confounded by organelle motion, which, by carrying localized tagged proteins in to and out of the plane of focus, could generate fluctuations that appear uncorrelated with the cell cycle (and most likely removed by the low-pass filtering). Cdc6 is a clear example of this phenomenon: no oscillations were discernible in the single plane measurements, but maximum z-projection data (Figure 3) demonstrate clearly that Cdc6 total abundance and spatial localization both oscillate during the cell cycle.

It is interesting to note that for the proteins that we observe as oscillatory (Clb2, Cln1, Cln2, Cdc15, Sic1, and Whi5), the periods and amplitudes have larger CVs than that of the inter-bud times (Table 2). This suggests that the cell-cycle control mechanism is buffered so that the timing of bud emergence and cell division is less noisy than the periodic fluctuations of individual components of the underlying control mechanism.

Net1 and Mcm1 are special cases because their mean fluorescence oscillates strongly during the cell cycle but at the same time they remain confined to specific cellular compartments, the nucleolus for Net1 and the nucleus for Mcm1. They call for establishing a distinction between protein abundance in concentration and total amount. Proteins that remain at a constant concentration through the cell cycle double in amount over one cell cycle period. When proteins are uniformly distributed in the cell, the protein concentration is well approximated by the average fluorescence of a sufficiently larger number of pixels. As a result, they are fairly insensitive to the limitations of the image segmentation algorithm. When a protein is localized in a small fraction of the cell volume, estimating the time-evolution of the concentration requires the division of the cell total fluorescence by an estimate of the cell volume, something that the image segmentation algorithm does not provide. Because the segmentation tends to delimit the contour of the mother cell and ignore the bud, the number of pixels used to average the fluorescence remains fairly constant throughout the cell cycle. Hence, the mean fluorescence measurement corresponds to the average concentration of Mcm1 (and Net1) in the mother portion of the cell (where the nucleus resides until nuclear division [41]), and the oscillations observed for Net1 and Mcm1 may reflect a doubling in amount in the mother cell, not necessarily in concentration averaged across mother and daughter. This interpretation could explain the discrepancy between data obtained by bulk measurement and by imaging. When we divide total fluorescence by total number of pixels in mother cell + bud (data not shown), we find that the oscillations in mean fluorescence of Net1 and Mcm1 are damped but not eliminated.

More fundamentally, it is not clear if average concentrations or total amounts are better indicators of biological activity. The notion of cellular concentration of a compound that remains confined to a very small fraction of the cell volume does not seem relevant. On the contrary, the possibility to combine localization and abundance data, as it is possible by imaging cytometry, may be a better telltale of protein dynamics than more traditional bulk measurements.

More generally, it is important to keep in mind that the relationship between protein abundance and fluorescence measurements remains poorly characterized *in vivo*. Very little information is available about the maturation and degradation rates of fluorescent proteins *in vivo*. It is not clear how these parameters are altered by fusion to another protein. It is also possible that the addition of the ∼27 kDa GFP moiety to the C-terminus of a cell cycle regulating protein may interfere with its function, as may be the case for Cdc20 and Sic1.

Contrary to more conventional measurement techniques that require extraction of proteins and RNAs from bulk cultures, time-lapse microscopy does not require synchronizing cells. It is therefore likely that the single cell measurements reported here better reflect the natural progression of the cell cycle than bulk measurements of synchronized cells that were used in the past. Not only could the synchronization process interfere with the cell cycle, but also bulk measurements could mask how each cell behaves throughout the division process. Also we are measuring a proxy for protein abundance, whereas other efforts to globally observe gene expression have focused mainly on transcription and mRNA levels [28], [29]. In order to gain a more comprehensive picture of cell cycle dynamics, future studies should combine single-cell measurement of mRNA [42] with single-cell proteomic data. Labeling multiple cell cycle genes with optically separable reporters would make it possible to estimate the correlation between the expression levels of different proteins.

## Materials and Methods

### Yeast Strains and Media

Each *S. cerevisiae* strain used in this study included GFP inserted as a C-terminal fusion downstream of a protein involved in cell-cycle control as described in [43]. All strains were purchased from Invitrogen (Carlsbad, CA).

All experiments were conducted using synthetic complete (SC) growth medium with 2% glucose at 30°C. Cell cultures were grown overnight from glycerol stocks, and prior to microscopic observation, the cells were verified to be in the exponential growth phase by measuring the OD_600_. Small volumes (∼3 µL) of liquid culture were added to a #1.5 microscope coverslip, and covered with a thin SC agar slab to limit cell motion.

### Genotyping

To verify the genotypes of the individual strains, they were first streaked on histidine dropout agar plates, colonies were selected and the genomic DNA extracted using the purelink™ genomic DNA extraction kit (Invitrogen, Carlsbad, CA) according to the manufacturer's instructions. The genomic DNA was then genotyped by polymerase chain reaction (PCR) using the corresponding CHK and R1 primer pairs from Huh et al. [43] in a master mix containing 1× HotStarTaq DNA polymerase master mix (Qiagen,Valencia, CA ) and 1.6 µM each primer in 30 µL volume. The amplification program was the following: 95 C for 1 min; 95 C for 30 s, 51 C for 30 s, 72 C for 2 min repeat 35×; 72 C for 10 min. The results were analyzed by agarose gel electrophoresis. The genotype is confirmed by a 2.8 kb band corresponding to the cassette GFPS65T-HIS3MX6 inserted at the expected genomic locus, whereas a band of 0.5 kb to 0.65 kb indicates a WT locus and the presence of the GFPS65T-HIS3MX6 cassette somewhere else in the genome.

### Fluorescence Microscopy

All images were collected on a DeltaVision microscope (Applied Precision, LLC, Issaquah, WA), which is equipped with an LED lamp for bright-field mode, and a 250 W Xenon lamp for fluorescence excitation. The DeltaVision has automated components, including the x-y-z translation stage, filter wheels, shutters, and is equipped with a CoolSNAP HQ camera (Photometrics, Tucson, AZ). A 60× phase contrast objective was used to collect both phase contrast and fluorescence images. A GFP filter-set with the excitation band centered at 470 nm (full-width of 40 nm) and emission band centered at 525 nm (full-bandwidth of 50 nm) was used to image GFP-expressing cells with an exposure time of 100 ms. Images were collected from 20 different regions for each cell line at 5-minute intervals for a total of 5 hours or until cell density became too great to permit identification. No significant photobleaching was observed in this period of time under these illumination conditions. The optimal focal plane for each region was found using the DeltaVision's built-in autofocus function in the Phase-contrast channel prior to collection of each time-point.

To ensure that the observed changes in localized concentration were not an artifact from an organelle moving in and out of focus, three-dimensional stacks consisting of 21 z-sections were obtained for each cell with a z-step size of 0.5 µm using the DeltaVision control software softWorx and the same filters and exposure times described above. Only 5 regions from each cell line could be observed with these conditions, due to the increased number of focal planes collected. We also noticed that the cells exhibited much longer inter-division times, which we believe is due to the increased amount of light exposure. Cell strains that did not contain GFP did not grow at all under these conditions (data not shown).

Movies of oscillating genes (Cln2 and Net1) are included in the online supplement (Movies S1, S2, S3, and S4). All images collected in the context of this work are available at https://sirion.vbi.vt.edu/yeast_GFP_data/.

### Image Processing

Phase-contrast images are segmented using custom software derived from Yeast Tree 1.6.3 [14]. The application relies on the MATLAB Image Processing toolbox. First, the function ‘imfill’ is used to flood-fill local minimum not connected to the image border, which fills in the center of the groups of cells. As each group of cells will have slightly different levels to which the flood-fill will rise, we then search the image histogram for intensities greater than the calculated background, taken from the border pixels, and with a frequency greater than the minimum cell area, generally set to 200 pixels. To keep only large groups of connected pixels, an erosion (built-in function ‘imerode’) is performed, removing the outermost pixels of a region and eliminating small groups of pixels.

The next step is to separate these groups into individual cells. This is done with another call to ‘imerode’ to cut the small necks that appear between touching cells. Once the cells are cut, the remaining connected regions are labeled with a call to the built-in function ‘bwlabel’, which identifies the individual cells and assigns each with a unique label. To finish, the cells are returned to their original sizes with a dilation (built-in function ‘imdilate’), which adds pixels around the edges of each cell.

After all frames in the time-series are segmented, the pixels making up each cell body are mapped to the previous frame by calculating the overlap (defined here as the ratio of the intersection of cell-body pixels to their union) of the current cell with the cells in the previous frame.

The three-dimensional stacks used for calculating localized concentration were reduced to 2D images by use of the maximum Z projection in MATLAB.

The image processing software used in this work is available in the online supplement (Software S1).

### Data analysis

In an effort to determine if each protein was oscillating with the cell cycle, we used spectral subtraction to remove noise [44]. Briefly, the mean noise spectrum, μ(*f*), is calculated by averaging the magnitudes of all wild type cells' Fourier spectra. For each cell of the 17 strains, an individualized filter, *H*(*f*), is then constructed such that
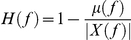
where |*X*(*f*)| is the magnitude of the Fourier transform, *X*(*f*) of the cell's time-course. The Fourier spectrum is then multiplied by the filter, and the inverse Fourier transform is taken to obtain the filtered time-domain data.

The resulting trajectories still contain high-frequency noise, as well as non-zero mean, so the individual cell trajectories are then de-trended by subtracting out the best-fit straight line from the time-course, and filtered using a low-pass Butterworth filter with a cut-off frequency set to 0.333 mHz (corresponding to a period of 50 min). The Butterworth filter is constructed such that the attenuation at the cut-off frequency is 3 dB. Peaks in the filtered data were then found by first locating local extrema, with the criteria that each peak (trough) must have a value > (<) 10% of the global maximum (minimum) for that cell, and that each pair of consecutive peaks (troughs) must be separated by 1 trough (peak). The period (in minutes) of each cycle was then estimated as the time between consecutive peaks, while the amplitude of each cycle was calculated as the difference between the first peak and the subsequent trough in that cycle.

The distribution of peak-to-peak times for each cell-line has been visualized in several ways. The 2-D histograms consist of 20 bins for each parameter. To obtain the boxes representing the 95^th^ percentile of cycles, we start by counting the number of cycles that fall into the bin representing the mean parameter values of the population. This box is then expanded 1 bin in each direction until 95% of the cells are contained in the box.

The standard deviation of the mean peak-to-peak time was calculated for each cell, in order to compare the variability from one cell cycle to the next in a single cell to the variability from cell to cell.

After applying the low-pass filter, all cells exhibit oscillation periods with mean values that are biologically feasible and the distributions of periods for proteins that are known to oscillate are not significantly different from the untagged (wild-type) strain. However the distributions of amplitudes for the various genes (Figure 4) are highly variable. Even the autofluorescence of the wild-type strain with no GFP tag shows low-amplitude oscillations (mean amplitude = 26 a.u., max amplitude less than ∼80 a.u.). To identify oscillatory proteins, we compared the amplitude distribution of each strain to the wild-type cells with a 2-sample, unequal variance t-test. At the 1% significance level, the amplitude distributions of following proteins could not have originated from the wild-type distribution: Cdc15, Clb2, Cln1, Cln2, Mcm1, Net1, Sic1, and Whi5.

Protein localization was quantified by first locating the pixel, *P*_max_, exhibiting the maximum fluorescence intensity, *I*_max_, within a given cell. Then each of the 8 pixels neighboring *P*_max_ was evaluated for whether its intensity was within the top 5% of the brightest pixels within the cell. If so, this pixel was added to the set containing *P*_max_. Then the nearest neighbors of the extended set were evaluated in like manner, until the set could be extended no further (or until the cell border was reached).

## Supporting Information

Appendix S1 **Assessment of data analysis algorithms on non-oscillating simulated cells.**
(PDF)

Movie S1 **Movie derived from Cln2 images (.mov format).**
(MOV)

Movie S2 **Movie derived from Cln2 images (.avi format).**
(AVI)

Movie S3 **Movie derived from Net1 images (.mov format).**
(MOV)

Movie S4 **Movie derived from Net1 images (.avi format).**
(AVI)

Software S1 **Image analysis software.**
(TAR)
