# Supplementary material for: Oscillatory Dynamics of Cell Cycle Proteins in Single Yeast Cells Analyzed by Imaging Cytometry
Source: PLoS One. 2011 Oct 26;6(10):e26272. doi: 10.1371/journal.pone.0026272 (PMC3202528; doi:10.1371/journal.pone.0026272)
Supplement: Appendix S1 — Assessment of data analysis algorithms on non-oscillating simulated cells. (PDF) [file pone.0026272.s001.pdf]

Online Supplement to Oscillatory dynamics of cell cycle proteins in single yeast cells analyzed by imaging cytometry

Assessment of data analysis algorithms on non-oscillating simulated cells

David A Ball; Julie Marchand; Magaly Poulet; William T. Baumann; Katherine C Chen; John J Tyson; Jean Peccoud

The data analysis was also performed on simulated trajectories of cell fluorescence that were produced from white noise, in order to determine if the filtering process would introduce any artifacts. Every time point from each of the 10,000 simulated cells was randomly taken from a Gaussian distribution with mean equal to the mean fluorescence of a wild-type cell (159.8 a.u.), and standard deviation equal to the pooled standard deviation of all wild-type cells (14.5 a.u.).

Budding events were also simulated, with the first budding event for each cell randomly chosen between 0 and 100 minutes with a uniform probability distribution. The timing of subsequent budding events was randomly chosen from a Gaussian distribution, such that the mean and standard deviation, σ, of the cell-cycle times coincided with the values for all real cells investigated in this study (mean = 84.9 min., σ = 23.5 min). An example simulated cell before and after filtering is shown in Figure S1 along with a wild-type cell for comparison.

Simulated cells were analyzed in the same way as experimental data leading to very different statistics for the simulated cells and untagged WT cells. This observation confirms that the fluctuations observed in WT cells are not the result of artifacts introduced by out data processing algorithm. The 2-D histogram of Period vs. Amplitude for a sample of 10,000 simulated cells is shown in Figure S2. The median amplitude of oscillation of the simulated cells after the application of spectral subtraction, and the low-pass filter was only 4.80 a.u. (WT was 11.83 a.u.). The oscillation periods of simulated cells was also significantly different from those of WT cells (and the other, tagged strains), with a median value of 60.00 min (WT was 84.98 min.). On average the peak of the simulated oscillations occurred at 1.4% of the cell-cycle (7.3% for WT) and the distribution of the simulated phases is much more uniform than that of the WT cells (Figure S3).

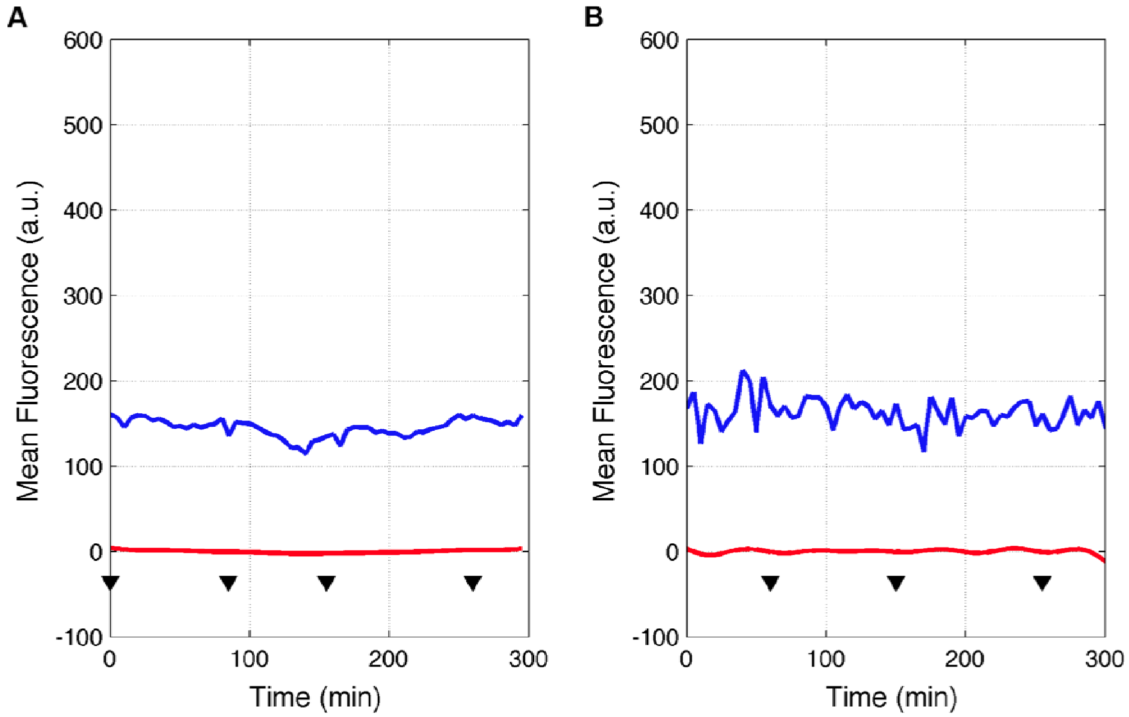

Figure S1: Comparison of mean cell fluorescence for a single real WT cell (A) and a simulated cell (B). The measured mean fluorescence is plotted (blue line) along with the filtered signal (red line) for a single cell simulated as purely white noise. Filtering removes all high-frequency noise, as well as the DC offset, leaving signals that have a zero mean. Budding events are indicated by black triangles.

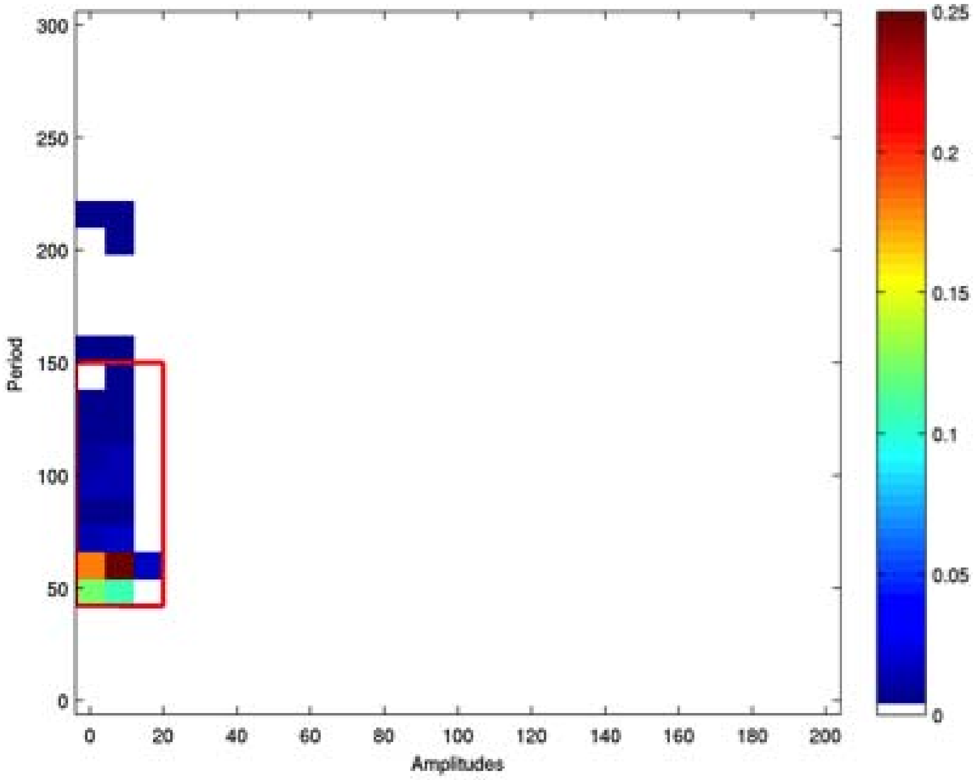

Figure S2: Distributions of amplitude and period estimates for 10,000 simulated cells. The red box encloses 95% of all cells. Low-pass Butterworth filtering removes all oscillations with non-physiological periods, which leaves only periods greater than 50 min. The color of each bin represents the fraction of cell cycles that fall in that bin, indicated by the color bar.

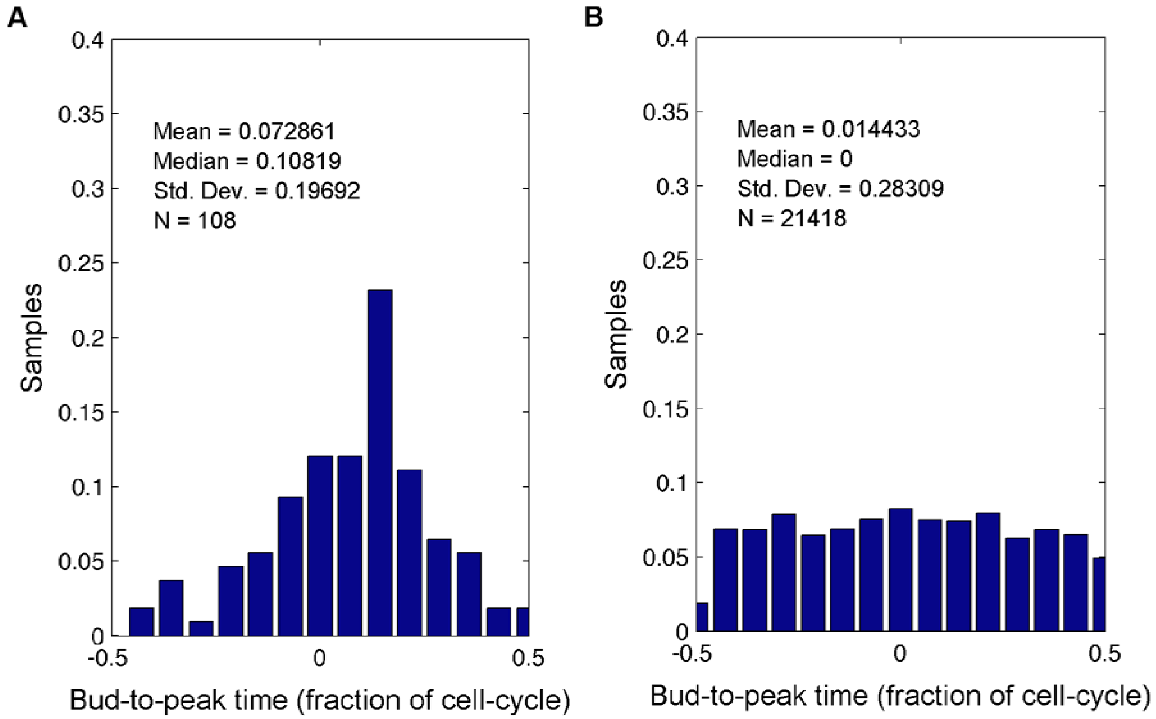

Figure S3: Comparison of the phase of peak expression in cell-cycle normalized time for the WT cells (A) and 10,000 simulated cells with a total of 21,418 cell cycles (B).
